# That Resolution of the Institute

**Published:** 1882-10

**Authors:** 


### THAT RESOLUTION OF THE INSTITUTE.

The last (September) number of the *Hahnemannian Monthly* contains an editorial effort to explain away the true meaning and *intent* of the resolution offered by Dr. Dudley, and adopted, "with one dissenting voice," by the few members of the Institute who were left at the tail-end of its recent session at Indianapolis, as the expression of the opinion and belief of the great American Institute of Homœopathy. A glance at the wording of the resolution will reveal what a careful perusal and study of it will but confirm, viz.: that it has not, nor was intended to have any reference whatever "to the question that now divides and distracts the allopathic denomination, viz.: the consultation question," unless at the expense of an admission of a greater subserviency to the "allopathic denomination" than has hitherto been conceded even by those who have been notoriously attempting to trade off the principles and precepts of Homœopathy for "old school recognition," for these many years past. The language of this resolution is explicit, as it is also pointed and direct, and "he that runneth" may readily comprehend its import without explanation or comment from its authors, instigators, or from those who were merely instrumental in its introduction and adoption.

That it contemplates just what it affirms, "*absolute* freedom of medical opinion and *unrestricted* liberty of professional action," cannot be doubted in the light of the history of Homœopathy for the past decade or two, as illustrated by the uniform course of action of that class of homœopathists who are responsible for its introduction and adoption. To the truth of this statement our journalism, society, presidential, and other addresses fully attest, as every reader of homœopathic journals and of our official papers must readily admit. So patent is this that it would be idle to specify, or to specifically comment upon this class of papers, surely not on those antecedent to the last and possibly most famous and outspoken of them all, viz.: the last presidential address before the American Institute of Homœopathy, in which these or similar views are advocated not only, but, if I remember correctly, an authoritative limit to the attenuations for therapeutic use by the members thereof, and of the profession at large, is also recommended and openly advocated therein, and this in the very face of an "*absolute* freedom of opinion and of *unrestricted* liberty of action" on the part of the same constituencies. "Consistency, thou art a jewel"—one, however, manifestly not possessed by these or other repudiators of law and principle and of long-established and well-recognized usages. This class of "*homœopathists*"—God save the mark!—want the fullest possible license, the one that will bring them nearest to "recognition" on the part of their allopathic brethren, and into the closest relations as to consultations, etc., with them, or with anybody and everybody possessing the legal evidence of a professional standing, *i. e.*, the plane upon which the New York State Allopathic Society now notoriously stands.

No effort on the part of the projectors of this villainously famous resolution can conceal its real purpose or the plainly exposed "cloven foot" of its authorship, wherever the body and brains of that hoof may lie concealed; or, if revealed, so diffusively as not *yet* to have assumed a definite shape, nor in the future, unless, indeed, the Institute in full session shall ratify and confirm this nefarious transaction.

The time, the occasion and all the surroundings and concomitants were well befitting this crowning act of treachery, this full surrender to the "allopathic denomination," and no contortion or interpretation of it can transform it into "a dignified, yet forcible, expression of the views of our school on this [consultation] question," except it be, as before remarked, on the basis of the most contemptible and abject subserviency to the behests of allopathy, in the interests of a falsely called liberal sentiment. This act was done, as it was initiated, by the dim, flickering light of the just expiring session of an institution in whose name it was thus basely and almost clandestinely accomplished; an act technically and legally possible, yet, nevertheless, *virtually* and damnably in violation of the *spirit* of all law and of equity itself, for the consummation of an act so vital, so fundamental as this, one so subversive of all the antecedent history and traditions of Homœopathy, as of the beliefs and practices of its ablest, wisest and oldest advocates and defenders.

The attempted defense of such an act is in full harmony and agreement with the methods of its introduction and passage. When the Institute ratifies and confirms this action it will cease to be the "American Institute of *Homœopathy,*" and will have become what the authors and *intelligent abettors* of it now are—Eclectic in principle and in practice. May the good Lord deliver and defend us and it from this and all similar perils!

*September 4th,* 1882. T. F. P.

